# Light-Induced Reversible Broadening of Ferroelectric Domain Walls

**DOI:** 10.1021/acs.nanolett.6c04817

**Published:** 2026-10-28

**Authors:** Fernando Rubio-Marcos, Jonathan Ordoñez-Pimentel, Adolfo Del Campo, Michel Venet, José E. García

**Affiliations:** † Department of Electroceramics, 119906 Instituto de Cerámica y Vidrio (ICV-CSIC), 28049 Madrid, Spain; ‡ Department of Physics, Universitat Politècnica de Catalunya, 08034 Barcelona, Spain; § Department of Physics, 67828 Universidade Federal de São Carlos, 13565-905 São Carlos, Brazil

**Keywords:** Domain walls, Light−matter interaction, Ferroelastic domains, Optical control, BaTiO_3_

## Abstract

Ferroelectric domain walls are active nanoscale interfaces whose functionality is governed by their internal structure rather than solely by their position. While optical stimuli are known to induce domain-wall motion, their impact on the spatial configuration of the domain-wall region remains unexplored. Here, we demonstrate that visible light induces a reversible and intensity-dependent broadening of ferroelectric domain walls in BaTiO_3_ crystals. Using confocal Raman microscopy, we resolve the evolution of the Raman-defined interfacial region and show that illumination transforms its spectroscopic signature from a relatively sharp boundary into an extended, graded interface. This broadening is associated with a continuous modification of the Raman response across the interfacial region and is fully reversible, with no evidence of irreversible structural changes. Our results reveal that domain walls can be dynamically reshaped by light, establishing optical excitation as a noncontact route to control the internal structure of ferroic interfaces and their associated functionalities.

## Graphical abstract

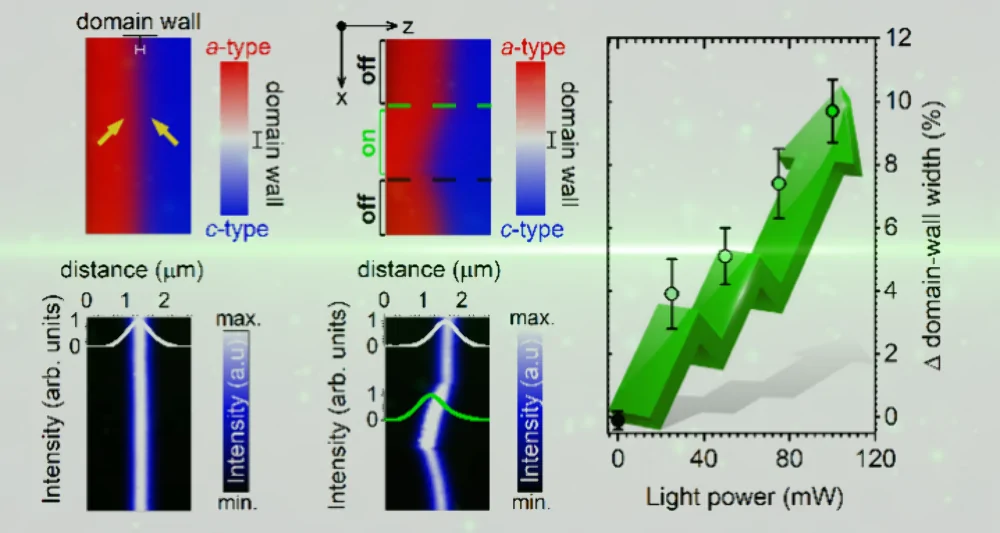

Ferroelectric domain walls are nanoscale interfaces separating regions of distinct polarization orientation, whose physical properties are increasingly recognized to transcend those of the surrounding bulk. Historically regarded as passive structural boundaries, domain walls are now understood as intrinsically active entities, capable of hosting distinct electronic, optical, and electromechanical functionalities arising from their reduced dimensionality and local symmetry breaking.^1–6^ This paradigm has led to a broader view of ferroic materials as assemblies of reconfigurable internal interfaces, in which domain walls act as functional elements governing the macroscopic response.

In this context, domain walls can be considered as emergent low-dimensional systems whose behavior parallels that of engineered interfaces in complex materials. Recent advances in van der Waals ferroelectrics have highlighted how polarization can arise from collective interfacial degrees of freedom, where stacking configurations, domain structures, and interlayer coupling define the functional response. Similarly, theoretical and computational studies have revealed that polarization in low-dimensional systems may adopt complex spatial textures, including vortical and chiral configurations, underscoring the richness of internal degrees of freedom associated with ferroic interfaces.^7^ These developments emphasize that the internal structure of ferroic interfaces is not fixed, but can evolve dynamically under external perturbations.^8^

A defining characteristic of ferroelectric domain walls is their finite spatial width, over which the polarization rotates continuously between adjacent domains. This interfacial region constitutes a gradient-driven state whose structure is highly sensitive to external stimuli, including electric fields, mechanical stress, temperature, and charge redistribution.^5,6^ Controlling these degrees of freedom is central to domain-wall nanoelectronics and to the development of functional devices based on reconfigurable interfaces. However, most experimental efforts have focused on domain-wall motion and position, while comparatively little attention has been paid to the dynamical control of their internal spatial profile.

Among the possible external stimuli, light provides a particularly attractive route to manipulate ferroic order. As a noncontact and spatially addressable perturbation, optical excitation can modify charge distributions, internal electric fields, and defect equilibria without the need for electrodes or mechanical coupling.^9–20^ Previous studies have demonstrated that illumination can drive domain-wall motion, reconfigure domain patterns, and even control macroscopic polarization states in a reversible manner.^12,21–23^ Despite these advances, whether optical excitation can directly renormalize the internal structure of domain walls, rather than merely displace them, remains an open question.

Addressing this problem is particularly relevant in light of emerging concepts in unconventional ferroelectricity, where polarization rearrangements may involve collective and correlated responses rather than independent local ionic displacements. Recent theoretical work has proposed that ferroelectric systems can transition toward regimes characterized by long ion displacements across unit cells, blurring the boundary between localized ferroelectric switching and collective transport phenomena.^24^ In parallel, optical excitation has been shown to generate nonlocal polarization responses mediated by propagating polaritonic excitations in ferroelectric materials.^25^ Although these phenomena are distinct from the local light-induced response investigated here, they illustrate the increasing complexity of polarization dynamics beyond conventional switching scenarios. In this broader context, ferroic interfaces, including domain walls, may undergo continuous transformations of their internal structure under external stimuli, rather than behaving as rigid boundaries.

Here, we demonstrate that visible light induces a reversible and intensity-dependent broadening of the Raman-resolved domain-wall region in BaTiO_3_ single crystals. Using confocal Raman microscopy, we resolve the spatial evolution of the interfacial Raman response and show that optical excitation transforms it from a relatively sharp interface into a progressively extended transition region. The broadening is accompanied by a continuous modification of the local spectral response, revealing a reversible light-induced reconfiguration of the Raman-resolved interfacial profile. These results establish optical excitation as an effective means to dynamically reshape ferroic interfaces at the nanoscale, opening new pathways for the control of interfacial functionalities in ferroic materials.

BaTiO_3_ single crystals (PI-KEM Ltd., UK), grown by top-seeded solution growth, were used in this study. The sample consists of a polished plate of dimensions 5 mm × 5 mm × 0.5 mm, oriented along the (100) crystallographic direction. The as-received crystal exhibits a stable ferroelastic multidomain configuration under ambient conditions, without the need for additional thermal or chemical treatment. Optical microscopy shows the presence of alternating domains separated by well-defined domain walls, forming a regular domain pattern across the surface (Figure 1a). The domain structure was further characterized by atomic force microscopy operated in topographic mode. Measurements performed along directions perpendicular to the domain walls show a periodic surface corrugation associated with the ferroelastic nature of the domains, with predictable out-of-plane domains exhibiting a measurable protrusion relative to in-plane domains (Figure 1b,c), thereby providing a topographic reference for correlating surface morphology with spectroscopic measurements. This combined structural characterization establishes a well-defined reference for the identification and analysis of domain-wall regions. High-resolution X-ray diffraction reveals the expected splitting of the pseudocubic reflections (Figure 1d), confirming both the single-crystal character and tetragonal phase of the sample. The relative intensity of the (002)/(200) degenerated reflections provides direct information on the volume fraction of ferroelastic domains with out-of-plane (labeled as *c*-domains) and in-plane (labeled as *a*-domains) orientations.^12^ Further details of the experimental procedures are provided in the Methods section of the Supporting Information.

**Figure 1:**
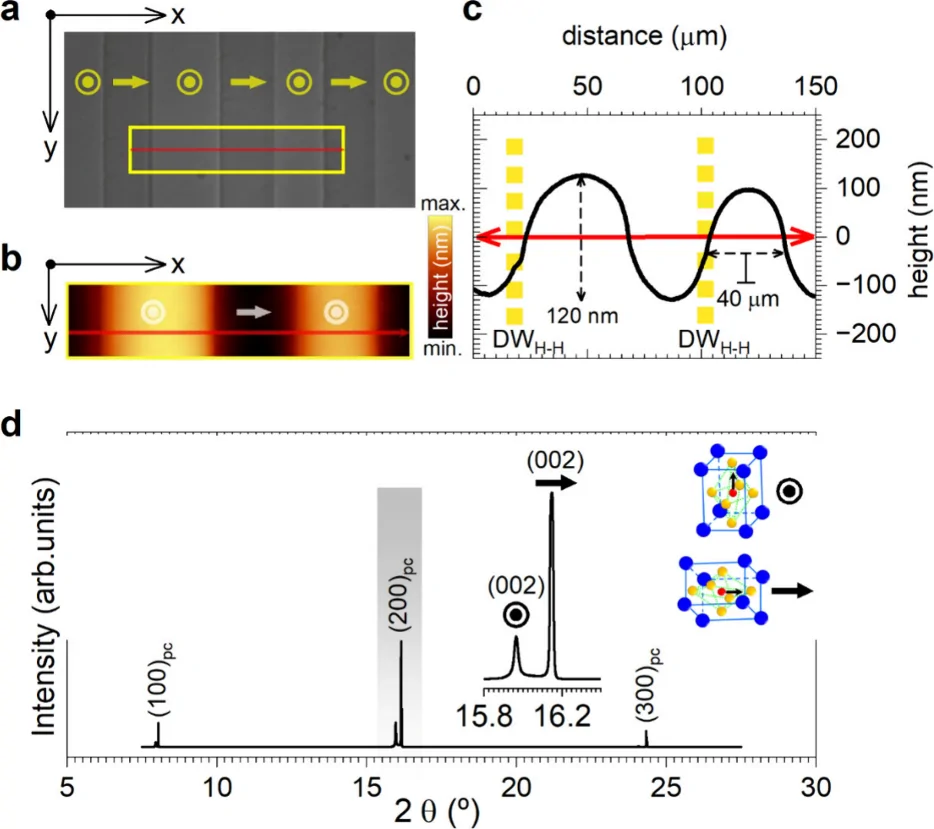
Optical, topographic, and X-ray characterization of the ferroelastic domain structure. (a) Optical microscopy image of the investigated region of the BaTiO_3_ single crystal, showing the alternating ferroelastic domain pattern at the crystal surface. The arrows and dots schematically indicate regions with predominantly in-plane and out-of-plane polarization orientations, respectively. (b) AFM topography image acquired in the region indicated in a yellow rectangle in panel (a), revealing the periodic surface corrugation associated with the ferroelastic domain configuration. (c) AFM topographic profile measured along the *x*-direction, perpendicular to the domain walls, as indicated by the red line in panels (a) and (b). The profile exhibits alternating protrusions and depressions associated with domains having out-of-plane and in-plane polarization components, respectively, reflecting the ferroelastic distortion linked to the different polarization orientations. (d) Synchrotron X-ray diffraction pattern of the BaTiO_3_ crystal. The splitting of the pseudocubic reflections confirms the tetragonal crystallographic symmetry and single-crystal nature of the sample. The relative intensity of the (002)/(200) doublet evidences the coexistence of ferroelastic domains with out-of-plane and in-plane polarization orientations, respectively.

Confocal Raman microscopy (WITec alpha300R) was employed as the primary technique to resolve the domain-wall structure. Measurements were carried out using a 532 nm excitation wavelength with a 100× objective (NA = 0.95), providing submicron lateral resolution and depth sensitivity. Raman maps were acquired with typical grids of 54 × 100 spectra and an integration time of 0.2 s per point, corresponding to a spatial sampling interval of approximately 50 nm in both directions. This sampling interval is smaller than the nominal diffraction-limited lateral resolution of the confocal Raman configuration (∼0.34 μm). Figure 2a shows a representative surface Raman map of the ferroelastic domain configuration, revealing alternating *a*- and *c*-domains separated by well-defined domain walls. Depth-resolved Raman mapping performed along a direction perpendicular to the surface trace of the wall reveals an apparent wall inclination of approximately 20° with respect to the sample surface in the experimental XZ coordinates (Figure 2b). This value represents an approximate geometrical estimate from the confocal image rather than a crystallographically calibrated wall-plane orientation, since no correction for refractive-index-induced axial scaling was applied. For quantitative profile analysis, the data sets were rotated by the complementary angle (∼70°) to align the apparent wall with the analysis reference frame before extracting profiles perpendicular to it (Supplementary Section S1). This transformation defines a consistent analysis direction but does not remove three-dimensional confocal-volume convolution or possible illumination-induced changes in wall geometry. The confocal configuration probes a finite volume below the surface, enabling a cross-sectional representation of the wall region. Based on this geometry, the effective domain-wall width was evaluated in the region indicated in Figure 2b and schematically defined in Figure 2c as the spatial extent of the diffuse transition zone separating adjacent domains.

**Figure 2:**
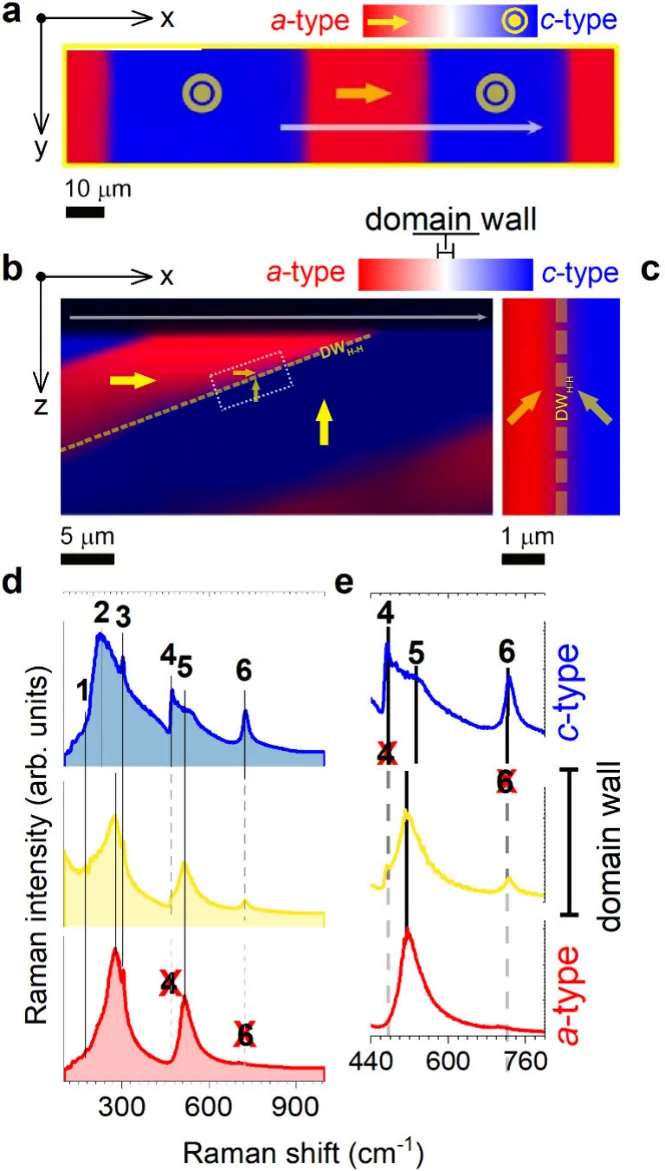
Confocal Raman characterization and operational definition of the ferroelectric domain-wall region. (a) Surface Raman map of the investigated region, showing the alternating ferroelastic domain pattern at the crystal surface. The red and blue contrast correspond to domains with predominantly in-plane (*a*-domains) and out-of-plane (*c*-domains) orientations, respectively, as schematically indicated above the map. (b) Depth-resolved Raman map acquired along the line indicated in panel (a), revealing the subsurface evolution of the ferroelastic domain structure and the inclined geometry of the domain wall beneath the crystal surface. The dashed rectangle marks the region used for the estimation of the effective domain-wall width. (c) Schematic representation of the operational definition of the domain-wall region. The effective wall width is defined as the spatial extent of the diffuse transition zone separating adjacent domains, corresponding to the region where mixed-domain spectral contributions coexist. (d) Representative Raman spectra corresponding to the three relevant regions: *a*-domain (red), *c*-domain (blue), and the intermediate mixed-domain region associated with the domain wall (yellow). The spectra exhibit characteristic differences in the relative intensity and suppression of specific Raman modes depending on the local polarization orientation. (e) Detailed view of the spectral region containing Raman modes 4–6. Modes around 463 cm^–1^ and 715 cm^–1^ are clearly present in *c*-domains and suppressed in *a*-domains, while remaining partially visible in the mixed-domain region. This behavior provides a direct spectroscopic fingerprint for identifying the domain-wall region and evaluating its effective width.

The Raman spectra were analyzed using a linear spectral unmixing procedure implemented in the WITec Control Plus software package. Representative reference spectra were first acquired from pure *a*- and *c*-domains (Figure 2d), while the characteristic Raman features used to distinguish the two ferroelectric domain types are highlighted in Figure 2e. Each Raman map consisted of 5400 spectra acquired over a 2.7 × 5 μm^2^ region using a 54 × 100 grid and an integration time of 0.2 s per spectrum, corresponding to a total acquisition time of approximately 18 min. Each measured spectrum was subsequently fitted as a linear combination of the *a*-domain and *c*-domain reference spectra over the analyzed spectral range (>150 cm^–1^), yielding the fractional spectral contributions of the *a*-domain (%a) and *c*-domain (%c) components at each spatial position. The resulting coefficients were used to reconstruct spatial maps of the relative contributions of the two domain spectral signatures. In particular, the Raman modes around 463 cm^–1^ and 715 cm^–1^, which are present in *c*-domains and suppressed in *a*-domains, provide a clear spectral fingerprint for distinguishing the domain type.^26,27^ The domain-wall region is therefore identified as a spatial zone where both contributions coexist, giving rise to a mixed spectral response (Figure 2e). Accordingly,%*a* ≈ 100% and %*c* ≈ 0% describes a spectrum dominated by the *a*-domain reference signature, whereas the opposite limit corresponds to the *c*-domain signature. Intermediate values indicate that both reference spectral signatures contribute to the measured spectrum.

To quantify the resulting Raman-resolved interfacial region, a wall-intensity metric was defined as DWi = %*a* × %*c*, where %*a* and %*c* correspond to the fractional contributions of the *a*- and *c*-domain spectral components obtained from the linear spectral unmixing procedure. The metric approaches zero within pure domains and increases when both spectral contributions are simultaneously present, thereby highlighting the mixed-domain region. The spatial distribution of DWi therefore provides an operational identification of the Raman-resolved boundary region separating adjacent ferroelectric domains. The effective wall width was determined from the full width at half-maximum (fwhm) of the DWi profile measured perpendicular to the wall. For each illumination power, in both the off and on states, ten DWi profiles were extracted at different positions along the domain-wall segment and independently fitted using a symmetric Gaussian function with a constant offset. The effective Raman-resolved interfacial width was obtained from the fitted fwhm, while its experimental variability was evaluated from the standard deviation of the ten extracted widths. The corresponding fitting uncertainties and complete statistical analysis are provided in Supplementary Section S3. This approach provides an effective operational measure of the domain-wall region, enabling direct comparison of its evolution under external perturbations.

Previous Raman studies on (100)-oriented BaTiO_3_ crystals identified, at some *a*–*c* boundaries, micrometer-scale regions termed *b*-domains, whose Raman response combined characteristics of the adjacent *a*- and *c*-domains and was interpreted as arising from a stress-relieving *a*/*c* subdomain structure.^10^ Subsequent work further showed that the density of such interfacial configurations influences the reversible, power-dependent optical response of BaTiO_3_.^28^ In the crystal investigated here, no spatially resolved *b*-domain regions comparable to those previously reported are observed within the experimental resolution; instead, the Raman maps show a direct *a*-to-*c* transition through the mixed-spectral region quantified by DWi. Nevertheless, because a *b*-domain-like unresolved subdomain structure would also produce mixed *a*/*c* spectral contributions, the DWi metric does not uniquely determine the microscopic origin of the Raman-resolved interfacial region. Redistribution of unresolved interfacial subdomains may therefore contribute to its illumination-induced evolution.

Once the domain-wall region was identified and quantitatively defined, external optical excitation was introduced to investigate possible modifications of its spatial profile. Illumination was provided by an external laser beam path independent of the Raman probe, ensuring that the probing conditions remained constant during the experiment. The complete optical configuration, including the independent Raman probing and external illumination beam paths, is schematically illustrated in Supplementary Section S2. The light intensity was controlled using a continuously variable neutral-density filter, allowing homogeneous attenuation without modifying the beam profile or spot size at the sample surface. The illumination wavelength (532 nm) lies below the bandgap of BaTiO_3_, ensuring sub-bandgap excitation conditions. The off condition corresponds to the absence of the external illumination beam while maintaining unchanged Raman probing conditions. Thus, any influence of the Raman probe is common to both off and on states, allowing the differential response to the additional external optical excitation to be evaluated. Raman maps were acquired under controlled off–on–off illumination sequences, enabling the direct observation of reversible changes in the domain-wall structure. The illumination was switched on and off at defined times during acquisition, so that different regions of each map correspond to successive stages of the illumination cycle. Because the complete map is acquired sequentially over approximately 18 min, with each illumination stage spanning about 6 min, the resulting profiles represent temporally sampled Raman-resolved configurations rather than instantaneous snapshots. Within each illumination stage, the widths extracted from successive profiles fluctuate around a stable mean value without showing a systematic temporal evolution. Nevertheless, the raster acquisition cannot exclude faster illumination-induced wall fluctuations or displacements occurring during individual line acquisition. The extracted width should therefore be regarded as a temporally averaged Raman-resolved interfacial width rather than as a direct measure of a static interfacial configuration.

The effect of illumination on the domain-wall structure is summarized in Figure 3, which shows the evolution of the same spatial region under controlled off–on–off illumination cycles. Under dark conditions, the *a*–*c* boundary appears as a sharp and well-defined interface separating the two ferroelastic domains. When illumination is applied, the domain wall undergoes a qualitative transformation: the boundary becomes progressively more diffuse, and the region of coexistence between *a*- and *c*-domains expands. At low illumination intensity, this effect is already visible as a widening of the transition region, accompanied by a displacement of the wall. As the light intensity increases, both effects become more pronounced, with the diffuse interfacial region extending over a larger spatial range. Importantly, the broadening occurs continuously, without the formation of additional domains, indicating that the wall evolves as a single, spatially extended interface rather than through discrete domain nucleation. This continuous evolution, in the absence of domain nucleation, further supports the interpretation of the domain wall as a deformable interface rather than a sequence of discrete switching events. This behavior indicates that the domain wall cannot be described as a rigid boundary under optical excitation, but rather as a dynamically reconfigurable interface whose spatial extent is continuously modified by light.

**Figure 3:**
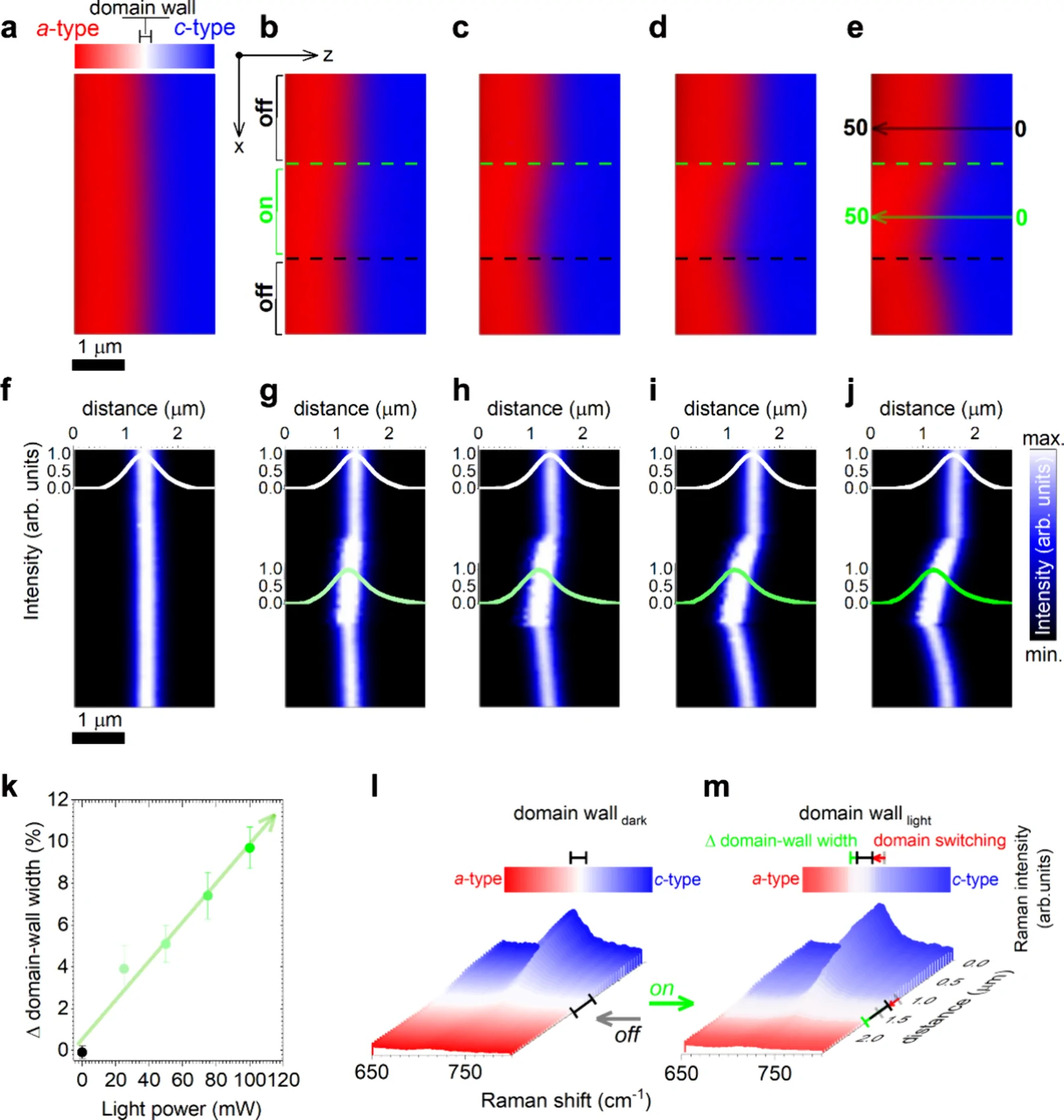
Light-induced reversible broadening of ferroelectric domain walls under visible-light illumination. (a–e) Confocal Raman maps acquired in the same spatial region during successive illumination cycles under 532 nm excitation, with illumination power increasing from 0 to 100 mW. Raman maps were sequentially acquired from left to right and top to bottom during scanning. The green dashed line marks the position at which the external illumination is switched on, while the black dashed line indicates the point at which the illumination is switched off again. The progressive modification of the diffuse interfacial region reveals a reversible light-induced broadening of the domain wall accompanied by a transient displacement of the domain boundary. (f–j) Raman-derived maps of the domain-wall region corresponding to the illumination sequence shown in panels (a–e). The profiles extracted across the wall are displayed in the corresponding panels, revealing a systematic increase of the effective domain-wall width under illumination. The broadening is fully reversible upon removal of the optical excitation. (k) Relative variation of the effective domain-wall width as a function of illumination intensity, showing a monotonic increase of the wall width with optical power. (l) Spatial sequence of Raman spectra acquired across the domain-wall region under dark conditions along the black line indicated in panel (e). The spectra show the transition between the characteristic responses of the *a*-type and *c*-type domains through a relatively narrow mixed-domain region. (m) Equivalent spectral sequence acquired under illumination (green line in panel (e)). Compared with the dark condition, the mixed-domain spectral response extends over a significantly larger spatial region, demonstrating that illumination induces not only a displacement of the domain boundary but also a reversible broadening of the Raman-resolved interfacial region.

This behavior is quantified in Figure 3k, where the relative change in the effective Raman-resolved interfacial width shows a monotonic increase as a function of illumination intensity. The broadening is approximately linear at low intensities and tends toward saturation at higher illumination levels. The effect is fully reversible: once the illumination is removed, both the position and the effective interfacial width of the domain wall recover their initial values, demonstrating the reversible nature of the light-induced response rather than a permanent structural modification.

Light-induced changes in domain-wall geometry may also contribute to the observed response. Photoinduced electronic screening has been shown to modify the inclination of 90° *a*/*c* domain walls in BaTiO_3_ thin films, while light-induced changes in the ferroelastic domain configuration of BaTiO_3_ have also been observed in strain-coupled heterostructures.^29,30^ In the present measurements, the independently determined wall inclination was taken into account through geometrical correction of the Raman data sets before extraction of the wall-width metric. Although a light-induced variation of the wall inclination cannot be independently resolved from the present measurements, the observed expansion of the mixed Raman spectral region demonstrates a reversible modification of the Raman-resolved interfacial profile under illumination. This evolution may reflect changes in the local electrostatic and stress environment of the domain-wall region. Light-induced redistribution and accumulation of charge carriers at the wall may modify the screening of bound polarization charge and thereby influence the local interfacial configuration. However, the present measurements do not uniquely determine whether the observed Raman broadening arises from a static modification of the polarization profile, redistribution of unresolved interfacial subdomains, dynamic wall fluctuations, or a combination of these effects.

Further insight into the nature of this broadening is obtained from the spatial evolution of the Raman spectra across the domain wall. Under dark conditions (Figure 3l), the transition between *a*- and *c*-domain spectral signatures occurs over a relatively narrow region. In contrast, under illumination (Figure 3m), the spectral evolution becomes more gradual, with mixed-domain features extending over a significantly larger distance. This gradual spectral evolution shows that illumination is accompanied by a reversible increase in the spatial extent over which mixed *a*- and *c*-domain spectral signatures are detected, in addition to the observed displacement of the Raman-resolved boundary.

Additional structural and thermal characterization (Figure 4) was performed to assess possible secondary effects of illumination. High-resolution synchrotron X-ray diffraction confirmed no measurable change in lattice parameters under illumination, indicating the absence of a structural phase transformation (Figure 4a,b). Importantly, the illuminated XRD measurement was performed at the maximum optical power used in the experiments. The experimental sensitivity to changes in *d*_002_ is ∼10^–4^ Å. Considering the reported room-temperature thermal-expansion coefficient of tetragonal BaTiO_3_ along the *c* axis (α_c_ = −6.7 × 10^–6^ K^–1^),^31^ this resolution implies that a temperature increase of approximately 7–8 K would produce a detectable displacement of the (002) reflection. No such displacement is observed. Independently, direct temperature measurements during illumination cycles show a maximum increase of only approximately 3 °C (Figure 4c,d). Together, these observations indicate that substantial photothermal heating is not the dominant origin of the observed light-induced response, although highly localized thermoelastic contributions cannot be completely excluded. The ability to continuously tune the spatial extent of the interfacial region establishes optical excitation as a powerful route to manipulate ferroic interfaces beyond simple domain reconfiguration, enabling dynamic control of their interfacial structure.

**Figure 4:**
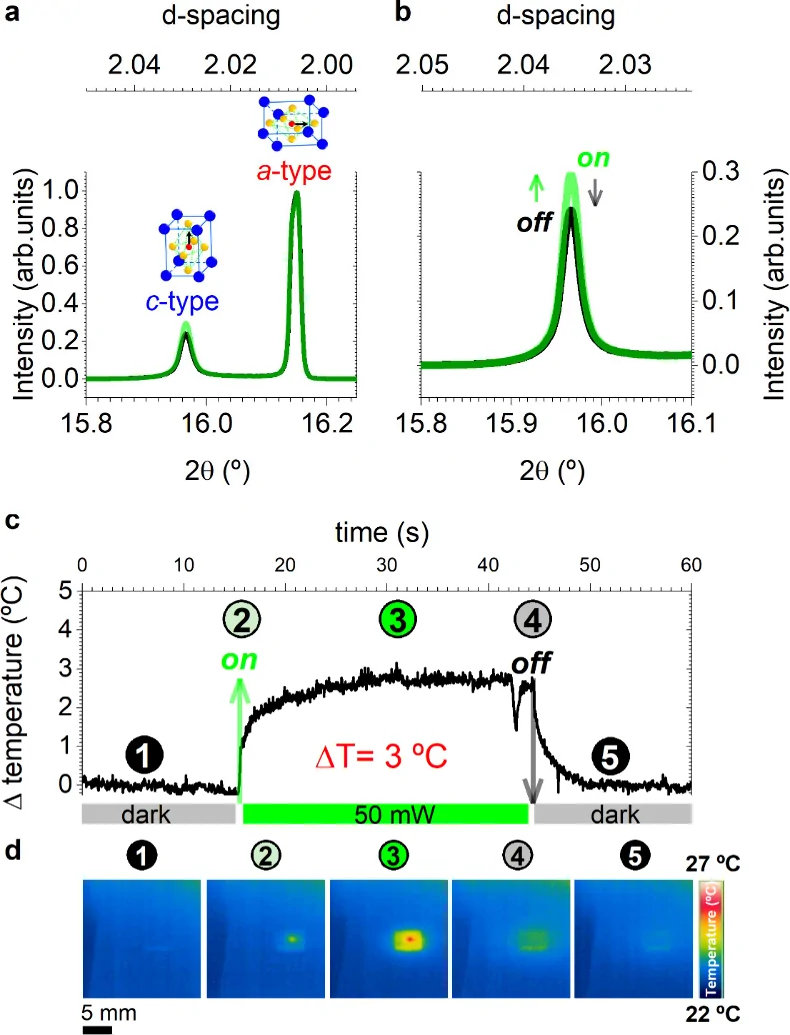
Structural and thermal response of the BaTiO_3_ crystal under visible-light illumination. (a) High-resolution synchrotron X-ray diffraction pattern of the BaTiO_3_ crystal showing the splitting of the pseudocubic reflections, consistent with the tetragonal crystallographic symmetry and ferroelastic domain configuration of the sample. (b) Evolution of the high-resolution XRD pattern of the (002)/(200) reflections during an off–on illumination cycle. Under illumination, the relative contribution of the reflection associated with domains having out-of-plane polarization orientation increases, indicating a reversible light-induced redistribution of ferroelastic domain populations and associated domain-wall motion. The absence of measurable peak shifts demonstrates that the lattice spacing remains unchanged during illumination, excluding light-induced structural phase transformations. (c) Time evolution of the sample temperature during an off–on–off illumination cycle. The temperature variation is plotted relative to room temperature, showing a maximum increase of approximately 3 °C under illumination. (d) Infrared thermal-camera images acquired at different stages of the illumination cycle corresponding to the numbered positions indicated in panel (c), showing the spatial distribution of the temperature across the sample surface during illumination and subsequent relaxation.

The proposed mechanism also suggests a direct functional consequence of the observed interfacial response. In charged ferroelectric domain walls, the carriers involved in compensating the bound polarization charge also govern the local electronic transport. Consequently, optical excitation may modify domain-wall conductivity through the coupled evolution of carrier population and the spatial extent of the interfacial region. Light-induced control of the domain-wall profile may therefore provide a noncontact route to tune the electrical functionality of conducting ferroelectric interfaces, opening a pathway toward optically addressable and reconfigurable domain-wall nanoelectronics.

In summary, we demonstrate that visible light provides a reversible and noncontact route to modify the Raman-resolved interfacial profile of ferroelectric domain walls. Illumination induces a controlled broadening of the effective interfacial region, corresponding to an extended mixed-spectral Raman response that evolves reversibly with optical excitation. This behavior reveals a dynamically reconfigurable domain-wall region whose spatial response can be continuously tuned by optical excitation. Because the local structure of charged domain walls is intimately coupled to their electronic properties, optical control of their spatial profile introduces a new degree of freedom for manipulating functional ferroic interfaces and provides a basis for light-reconfigurable domain-wall devices.

## Supplementary Material



## Notes

Experimental methods for sample preparation, atomic force microscopy, synchrotron X-ray diffraction, confocal Raman microscopy, optical excitation, illumination protocol, and thermal characterization; determination of the domain-wall geometry and geometrical correction (Figure S1); experimental geometry for domain-wall mapping under illumination (Figure S2); determination and statistical analysis of the Raman-resolved interfacial width (Figure S3 and Table S1); Raman-active modes and domain selectivity in tetragonal BaTiO_3_ (Table S2) (PDF)

## References

[1] Meier D., Valanoor N., Zhang Q., Lee D. (2021). Domains and Domain
Walls in Ferroic Materials. J. Appl. Phys..

[2] Schultheiß J., Picht G., Wang J., Genenko Y., Chen L., Daniels J., Koruza J. (2023). Ferroelectric
Polycrystals: Structural
and Microstructural Levers for Property-Engineering via Domain-Wall
Dynamics. Prog. Mater. Sci..

[3] Catalan G., Seidel J., Ramesh R., Scott J. F. (2012). Domain
Wall Nanoelectronics. Rev. Mod. Phys..

[4] Nataf G. F., Guennou M., Gregg J. M., Meier D., Hlinka J., Salje E. K. H., Kreisel J. (2020). Domain-Wall
Engineering and Topological
Defects in Ferroelectric and Ferroelastic Materials. Nat. Rev. Phys..

[5] Meier D., Selbach S. M. (2022). Ferroelectric Domain Walls for Nanotechnology. Nat. Rev. Mater..

[6] Sharma P., Moise T. S., Colombo L., Seidel J. (2022). Roadmap for
Ferroelectric
Domain Wall Nanoelectronics. Adv. Funct. Mater..

[7] Di
Rino F. N., Verhagen T. (2026). Switchable Topological Polar Textures
in Freestanding Ultrathin Ferroelectric Oxides. Nano Lett..

[8] Yan Y., Wu M. (2026). Transition from Conventional
Ferroelectricity to Ion-Conduction-Like
Ferroelectricity. Nano Lett..

[9] Yang S. Y., Seidel J., Byrnes S. J., Shafer P., Yang C.-H., Rossell M. D., Yu P., Chu Y.-H., Scott J. F., Ager J. W., Martin L. W., Ramesh R. (2010). Above-Bandgap
Voltages
from Ferroelectric Photovoltaic Devices. Nat.
Nanotechnol..

[10] Rubio-Marcos F., Del Campo A., Marchet P., Fernández J. F. (2015). Ferroelectric
Domain Wall Motion Induced by Polarized Light. Nat. Commun..

[11] McGilly L. J., Yudin P., Feigl L., Tagantsev A. K., Setter N. (2015). Controlling Domain Wall Motion in
Ferroelectric Thin
Films. Nat. Nanotechnol..

[12] Rubio-Marcos F., Ochoa D. A., Del Campo A., García M. A., Castro G. R., Fernández J. F., García J. E. (2018). Reversible
Optical Control of Macroscopic Polarization in Ferroelectrics. Nat. Photonics.

[13] Yang M.-M., Alexe M. (2018). Light-Induced Reversible Control
of Ferroelectric Polarization in
BiFeO_3_. Adv. Mater..

[14] Akamatsu H., Yuan Y., Stoica V. A., Stone G., Yang T., Hong Z., Lei S., Zhu Y., Haislmaier R. C., Freeland J. W., Chen L.-Q., Wen H., Gopalan V. (2018). Light-Activated
Gigahertz Ferroelectric Domain Dynamics. Phys.
Rev. Lett..

[15] Bai Y., Vats G., Seidel J., Jantunen H., Juuti J. (2018). Boosting Photovoltaic
Output of Ferroelectric Ceramics by Optoelectric Control of Domains. Adv. Mater..

[16] Vats G., Bai Y., Zhang D., Juuti J., Seidel J. (2019). Optical Control of
Ferroelectric Domains: Nanoscale Insight into Macroscopic Observations. Adv. Opt. Mater..

[17] Guo J., Chen W., Chen H., Zhao Y., Dong F., Liu W., Zhang Y. (2021). Recent Progress in Optical Control of Ferroelectric
Polarization. Adv. Opt. Mater..

[18] Dwij V., De B. K., Rana S., Kunwar H. S., Yadav S., Sahu S. R., Venkatesh R., Lalla N. P., Phase D. M., Shukla D. K., Sathe V. G. (2022). Optical
Control of Domain Configuration
Through Light Polarization in Ferroelectric BaTiO_3_. Phys. Rev. B.

[19] Ordoñez-Pimentel J., Venet M., Ochoa D. A., Rubio-Marcos F., Fernández J. F., García J. E. (2022). Light-Driven Motion of Charged Domain
Walls in Isolated Ferroelectrics. Phys. Rev.
B.

[20] Eliseev E. A., Morozovska A. N., Vysochanskii Y. M., Yurchenko L. P., Gopalan V., Chen L. Q. (2024). Light-Induced
Transitions of Polar
State and Domain Morphology of Photoferroelectric Nanoparticles. Phys. Rev. B.

[21] Rubio-Marcos F., Páez-Margarit D., Ochoa D. A., Del Campo A., Fernández J. F., García J. E. (2019). Photo-Controlled Ferroelectric-Based
Nanoactuators. ACS Appl. Mater. Interfaces.

[22] López-Sánchez J., Del Campo A., Quesada A., Rivelles A., Abuín M., Sainz R., Sebastiani-Tofano E., Rubio-Zuazo J., Ochoa D. A., Fernández J. F., García J. E., Rubio-Marcos F. (2024). Concomitant Light-Reversible Magnetic Response in Multiferroic
Oxide Heterostructures for Multiphysics Applications. ACS Appl. Mater. Interfaces.

[23] Ochoa D. A., Menéndez E., López-Sánchez J., Del Campo A., Ma Z., Spasojevic I., Fina I., Fernández J. F., Rubio-Marcos F., Sort J., García J. E. (2024). Reversible
Optical Control of Magnetism
in Engineered Artificial Multiferroics. Nanoscale.

[24] Yan Y., Wu M. (2026). Transition from Conventional
Ferroelectricity to Ion-Conduction-Like
Ferroelectricity. Nano Lett..

[25] Henstridge M., Först M., Rowe E., Fechner M., Cavalleri A. (2022). Nonlocal Nonlinear
Phononics. Nat. Phys..

[26] Shiratori Y., Pithan C., Dornseiffer J., Waser R. (2007). Raman Scattering Studies
on Nanocrystalline BaTiO_3_ Part I - Isolated Particles and
Aggregates. J. Raman Spectrosc..

[27] Shiratori Y., Pithan C., Dornseiffer J., Waser R. (2007). Raman Scattering Studies
on Nanocrystalline BaTiO_3_ Part II - Consolidated Polycrystalline
Ceramics. J. Raman Spectrosc..

[28] Páez-Margarit D., Rubio-Marcos F., Ochoa D. A., Del Campo A., Fernández J. F., García J. E. (2018). Light-Induced Capacitance Tunability
in Ferroelectric Crystals. ACS Appl. Mater.
Interfaces.

[29] Ahn Y., Everhardt A. S., Lee H. J., Park J., Pateras A., Damerio S., Zhou T., DiChiara A. D., Wen H., Noheda B., Evans P. G. (2021). Dynamic Tilting of Ferroelectric
Domain Walls Caused by Optically Induced Electronic Screening. Phys. Rev. Lett..

[30] Bagri A., Jana A., Panchal G., Chowdhury S., Raj R., Kumar M., Gupta M., Reddy V. R., Phase D. M., Choudhary R. J. (2023). Light-Controlled Magnetoelastic Effects in Ni/BaTiO_3_ Heterostructures. ACS Appl. Mater.
Interfaces.

[31] Joho P. (1964). Präzisionsmessung
der Gitterkonstanten und des Ausdehnungskoeffizienten von Bariumtitanat. Z. Kristallogr..

