# Supplementary material for: Light-Induced Reversible Broadening of Ferroelectric Domain Walls
Source: Nano Lett. 2026 Oct 28;26(44):15490–6. doi: 10.1021/acs.nanolett.6c04817 (PMC13761696; doi:10.1021/acs.nanolett.6c04817)
Supplement: Supplementary file 1 [file nl6c04817_si_001.pdf]

SUPPLEMENTARY INFORMATION

Light-Induced Reversible Broadening of Ferroelectric Domain Walls

Fernando Rubio-Marcos[1], Jonathan Ordoñez-Pimentel[2,3], Adolfo Del Campo[1],

Michel Venet[3], and José E. García[2]

[1] Department of Electroceramics, Instituto de Cerámica y Vidrio (ICV-CSIC), 28049 Madrid, Spain

[2] Department of Physics, Universitat Politècnica de Catalunya, 08034 Barcelona, Spain

[3] Department of Physics, Universidade Federal de São Carlos, 13565-905 São Carlos, Brazil

Table of contents

S0. Methods 2

S1. Determination of the domain-wall geometry 5

S2. Experimental geometry for domain wall mapping under illumination................. 6

S3. Statistical analysis of the Raman-resolved interfacial width 8

S4. Raman-active modes and domain selectivity in tetragonal $BaTiO_3$ 122

S0. Methods

Sample preparation. $BaTiO_3$ (BTO) single crystals were supplied by PI-KEM Ltd. (UK) and grown by the top-seeded solution growth (TSSG) method. The crystals were prepared as (100)-oriented plates with dimensions of 5 × 5 × 0.5 mm^3. The samples were mechanically polished using 1 µm diamond paste and subsequently cleaned with acetone and ethanol prior to characterization. No thermal or chemical etching treatments were applied in order to avoid introducing additional surface roughness or topographic modifications that could affect the optical measurements.

Atomic force microscopy. Atomic force microscopy (AFM) measurements were performed using the AFM module integrated into a WITec alpha300RA confocal Raman microscope. Gold-coated silicon cantilevers (NSG30, NT-MDT, Russia), with a nominal tip radius of approximately 10 nm, a spring constant of 40 N m^{-1}, and a resonance frequency of 288 kHz, were used. Topographic images were acquired in non-contact mode over scan areas of up to 150 × 75 μm^2, using 256 scan lines and 512 points per line. The feedback setpoint was maintained at 50% of the free oscillation amplitude to ensure stable topographic imaging.

X-ray diffraction. High-resolution X-ray diffraction measurements were performed at the SpLine CRG BM25 beamline of the European Synchrotron Radiation Facility (ESRF, Grenoble, France). The X-ray wavelength was set to 0.5636 Å (22 keV), with an energy resolution of $\Delta E/E \sim 10^{-4}$. The beam size at the sample was adjusted to approximately 0.5 × 0.5 mm^2. Measurements were carried out using a custom-designed high-resolution θ–2θ diffractometer equipped with a multi-analyser detector stage. The detector system incorporates ten NaI(Tl) scintillation point detectors operating in parallel, each equipped with a Ge(111) single-crystal analyser in a θ–2θ configuration. This configuration provides high angular resolution while maintaining relatively short acquisition times through simultaneous parallel detection. Instrument calibration and wavelength refinement were performed using a silicon standard. The crystal was mounted in a horizontal flat-plate geometry and the diffractometer was aligned to position the incident beam on the selected region of the sample. XRD patterns were recorded over the angular range 7.5°–27.5° in 2θ, using a step size of 0.0025° and an acquisition time of 1 s per step. Under these conditions, changes in the interplanar spacing of the order of 10^{-4} Å could be detected. Additional measurements restricted to selected diffraction peaks were performed to verify data reproducibility, using the same angular step and acquisition time. For measurements under illumination, a 532 nm laser diode (Thorlabs, Inc.) was coupled to the setup. Measurements were performed at 100 mW of light power. The illumination spot was adjusted to approximately 1 ×

1 mm^2 using an iris, and the irradiation time was controlled electronically. XRD patterns were recorded during successive light-on and light-off conditions to evaluate the reversibility of the response.

Confocal Raman microscopy. Ferroelectric domain characterization and Raman mapping were performed using a WITec alpha300RA confocal Raman microscope equipped with a 532 nm excitation laser and a 100× objective lens with a numerical aperture of 0.95. The nominal diffraction-limited lateral resolution of this optical configuration is approximately 0.34 µm. Two complementary Raman mapping geometries were used. Surface domain distributions were characterized using XY Raman maps acquired parallel to the sample surface, whereas the subsurface domain-wall geometry was independently investigated using depth-resolved XZ Raman maps. These measurements showed that the investigated *a*-*c* domain wall was inclined by approximately 20° with respect to the sample surface. For quantitative analysis, the Raman datasets were geometrically corrected using this independently measured inclination. The maps were rotated before profile extraction so that the domain-wall profiles were evaluated perpendicular to the local wall orientation, thereby minimizing projection effects associated with the static wall inclination. Large-area XY Raman maps typically covered 150 × 75 µm^2 and consisted of 150 × 75 spectra acquired with an integration time of 0.1 s per spectrum. The XZ depth-resolved Raman maps were acquired using the same excitation wavelength and objective configuration. For quantitative analysis of the interfacial region, high-resolution Raman maps covering 2.7 × 5 µm^2 were acquired around the domain wall. Each map consisted of 5400 spectra recorded on a 54 × 100 pixel grid with an integration time of 0.2 s per spectrum. This corresponds to a spatial sampling interval of approximately 50 nm in both directions and a total acquisition time of approximately 18 min per map. The resulting datasets were used for spectral-unmixing analysis and determination of the Raman-derived domain-wall metric (DWi). The DWi was obtained by spectral unmixing of the characteristic Raman signatures of the adjacent *a*- and *c*-domains. The Raman modes used for domain identification and their corresponding literature assignments are summarized in Table S2 of Section S4. The resulting DWi parameter describes the spatial evolution of the mixed a/c Raman response across the interface. Accordingly, the FWHM extracted from the DWi profile is interpreted as an effective Raman-resolved interfacial width, rather than as a direct measurement of the intrinsic nanometric polarization-gradient width of the ferroelectric domain wall. The procedure used to extract the interfacial width and evaluate its experimental variability and fitting uncertainty is described in Section S3.

Optical excitation configuration. Optical excitation was provided by an external laser-diode beam path independent of the Raman excitation source. Both the Raman probe and the external excitation beam operated at a wavelength of 532 nm but served different experimental purposes. The Raman laser integrated into the confocal microscope was maintained under unchanged acquisition conditions throughout the measurements and served as the spectroscopic probe. The external laser diode (Thorlabs, Inc.) was used to induce the optical perturbation, with its incident power varied between 0 and 100 mW. The polarization state and optical geometry of the external illumination beam were kept unchanged throughout the power-dependent experiments, with optical power as the controlled experimental variable. The external illumination setup consisted of a laser-diode mount, micropositioning stages, a continuously variable neutral-density (ND) filter, an iris, a power meter, a plano-convex lens, and the sample holder. The ND filter was used during power-dependent measurements, whereas the power meter was inserted into the optical path for calibration and verification of the incident optical power. The illumination beam was expanded to a diameter of approximately 2 mm at the sample surface, ensuring homogeneous illumination over the entire region investigated by Raman microscopy. The incident optical power was controlled using the continuously variable ND filter. Because the beam diameter at the filter position (~1 mm) was substantially smaller than the characteristic length scale of the optical-density gradient, attenuation was effectively uniform across the beam cross-section, allowing the incident power to be varied without appreciable modification of the beam profile, divergence, or spot size at the sample surface.

Illumination protocol. The reversibility of the optical response was investigated using off-on-off illumination sequences. Throughout the experiment, the Raman probe laser remained unchanged and therefore formed part of the baseline acquisition conditions. The off state thus corresponds to Raman acquisition in the absence of external illumination, whereas the on state corresponds to Raman acquisition under simultaneous external optical excitation. The Raman maps were acquired continuously over a total period of approximately 18 min. The off-on-off sequence consisted of approximately 6 min without external illumination, followed by approximately 6 min under illumination and a final approximately 6 min after the external illumination was switched off. Because the Raman map was acquired sequentially, the resulting profiles represent temporally sampled Raman-resolved configurations rather than instantaneous domain-wall snapshots. Within each illumination stage, successive profiles were used to evaluate the stability and variability of the extracted Raman-resolved interfacial width.

Thermal Characterization: The thermal response of the $BaTiO_3$ crystal under illumination was monitored using a FLIR T440 infrared camera. Data acquisition and processing were performed using FLIR Tools+ software. The surface temperature was recorded during off-on-off illumination cycles under the illumination conditions used in the optical experiments.

S1. Determination of the domain-wall geometry

Fig. S1 summarizes the procedure used to determine the three-dimensional domain-wall geometry and to define the reference frame employed for quantitative analysis. The depth-resolved XZ Raman map shown in Supplementary Fig. S1a reveals that the investigated a-c domain wall is inclined by approximately 20° with respect to the sample surface. Because quantitative analysis requires profiles extracted perpendicular to the local wall orientation, the experimental datasets were geometrically corrected using the apparent wall orientation determined from the XZ confocal Raman map. As illustrated in Supplementary Fig. S1b, the maps were rotated by approximately 70°, corresponding to the complementary angle of the apparent 20° inclination relative to the sample surface. This procedure removes the geometrical projection associated with the static wall inclination and establishes a common reference frame for comparison of the different datasets.

The resulting geometrically corrected domain-wall map is shown in Supplementary Fig. S1c. Profiles were subsequently extracted perpendicular to the corrected wall orientation and used to determine the effective Raman-resolved interfacial width. Supplementary Fig. S1d provides a three-dimensional schematic of the ferroelastic domain configuration, illustrating the relationship between the actual inclined domain wall, the Raman probing geometry, and the reference frame adopted for quantitative analysis.

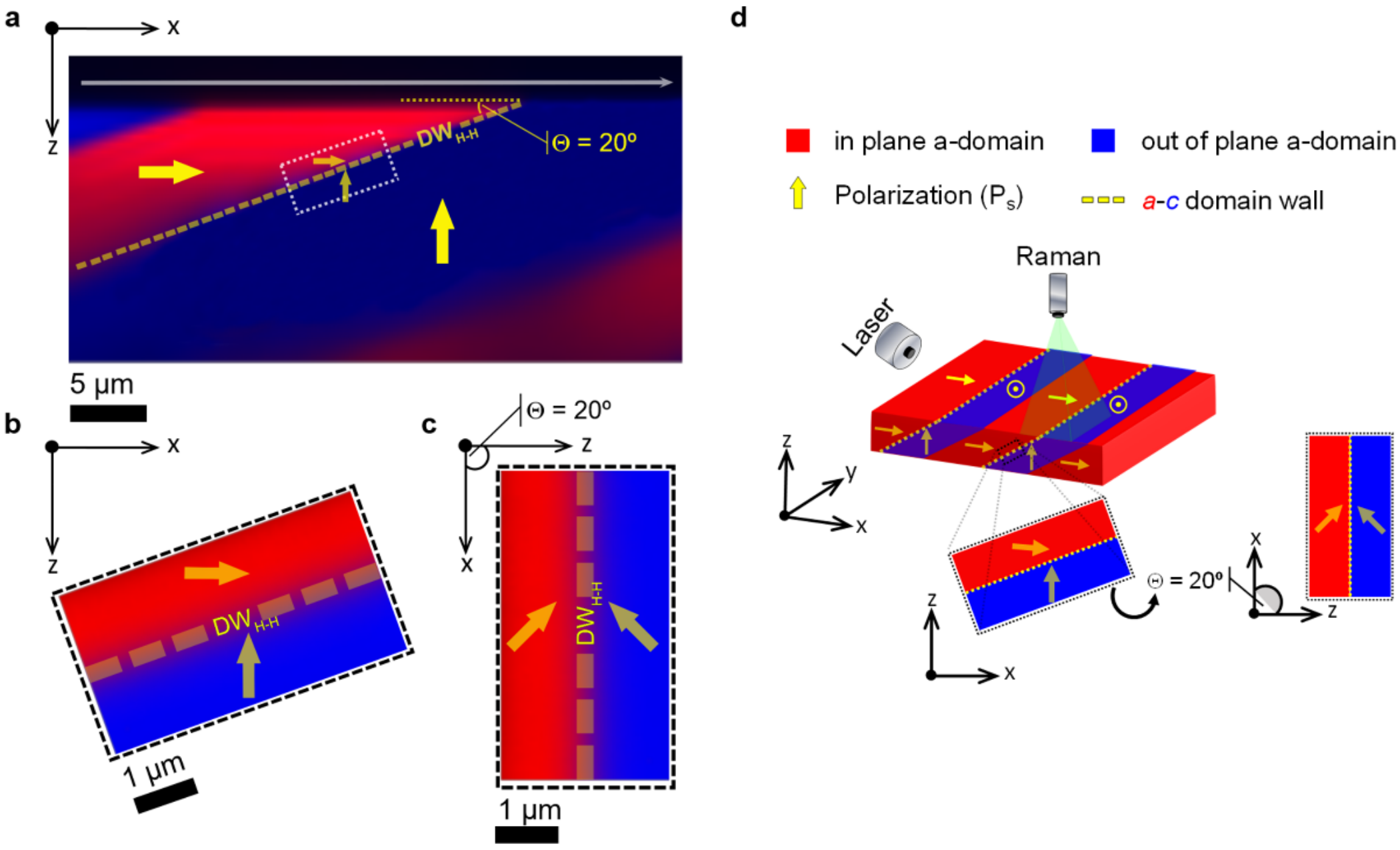

Figure S1 | Determination of the domain-wall geometry and geometrical correction applied for quantitative analysis. a, Depth-resolved XZ Raman map showing the ferroelastic *a-c* domain configuration in $BaTiO_3$. The domain wall is inclined by approximately 20° with respect to the sample surface. The dashed rectangle indicates a representative region used for wall-profile analysis. **b**, Geometrical correction of the XZ Raman map using the independently measured wall inclination. The map was rotated by the corresponding complementary angle to align the domain wall with the analysis reference frame. **c**, Raman-derived domain-wall map after geometrical correction. Profiles were extracted perpendicular to the wall orientation to determine the effective Raman-resolved interfacial width. **d**, Three-dimensional schematic illustrating the relationship between the actual ferroelastic domain structure, the inclined domain wall, the Raman probing geometry, and the reference frame used for quantitative analysis.

S2. Experimental geometry for domain wall mapping under illumination

Fig. S2 summarizes the experimental configuration used for confocal Raman mapping under external illumination. As shown in Fig. S2a, Raman measurements were performed on *a-c* domain walls previously identified by depth-resolved XZ Raman mapping. The schematic illustrates the relative orientation of the adjacent ferroelastic domains, the Raman probing beam, and the external illumination beam used during the experiments.

The XZ Raman measurements showed that the investigated domain wall is inclined by approximately $\theta_{DW} \approx 20°$ with respect to the sample surface. Prior to quantitative analysis, the selected regions were

therefore geometrically corrected using this independently measured inclination, as illustrated in Fig. S2b. This procedure aligns the wall with the analysis reference frame and ensures that the extracted profiles are evaluated perpendicular to the local wall orientation.

For the illumination experiments, Raman maps were acquired continuously over a total period of approximately 18 min. The external illumination followed an off-on-off sequence consisting of approximately 6 min without external illumination, 6 min under illumination, and a final 6 min after the illumination was switched off. Because the Raman map was acquired sequentially, the resulting profiles represent temporally sampled Raman-resolved configurations rather than instantaneous domain-wall snapshots. This protocol enables direct comparison of the interfacial response before, during, and after external illumination under otherwise unchanged Raman probing conditions.

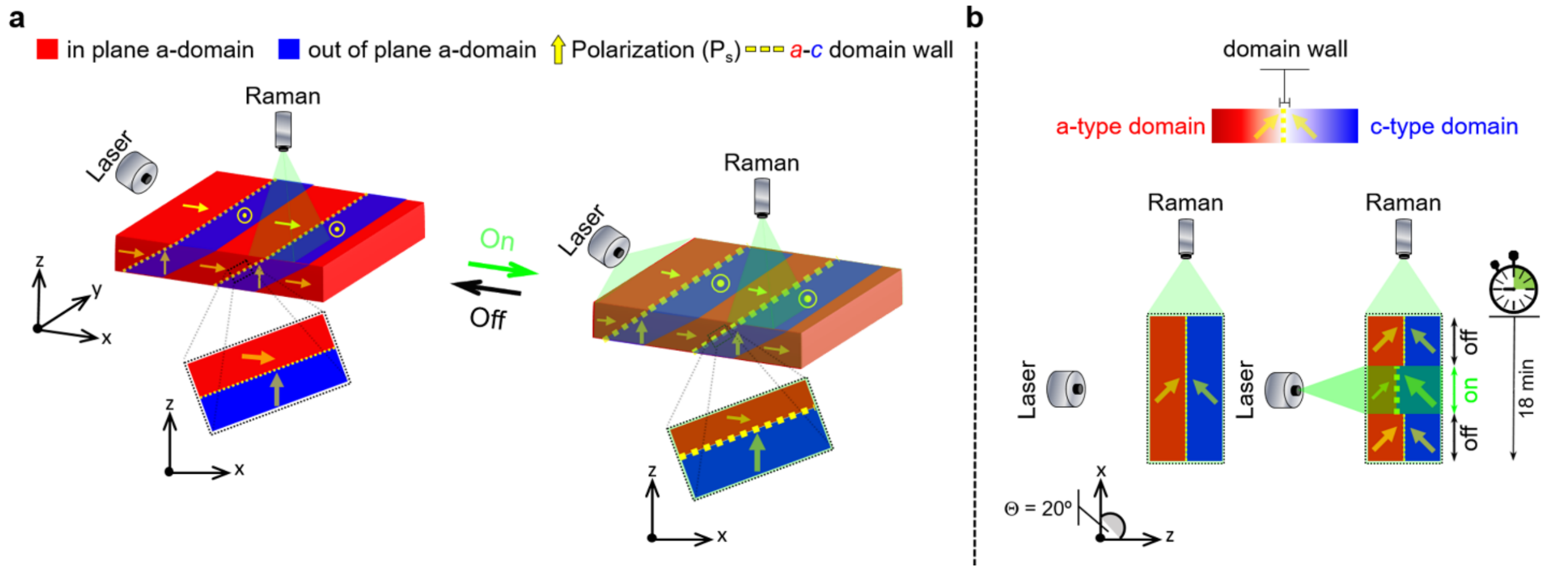

Figure S2 | Experimental geometry for confocal Raman mapping under illumination. a, Three-dimensional schematic of the ferroelastic *a*- and *c*-domain configuration in the $BaTiO_3$ single crystal. The dashed yellow line indicates the *a*–*c* domain wall. The Raman probing beam and the external illumination beam are shown together with the polarization directions of the adjacent domains. **b**, Geometrical correction and optical measurement configuration used for domain-wall analysis. The independently determined wall inclination, $\theta_{DW} \approx 20°$, is used to define the analysis reference frame, and the off-on-off sequence indicates the temporal organization of the illumination experiment.

S3. Statistical analysis of the Raman-resolved interfacial width

The Raman-resolved interfacial width was determined from two-dimensional domain-wall index (DWi) maps obtained by spectral unmixing of the Raman response. At each spatial position, the DWi was calculated as the product of the relative *a*- and *c*-domain spectral contributions (%*a* × %*c*). Consequently, DWi approaches zero within regions dominated by either domain configuration and reaches its highest values where the Raman signatures of both domains coexist. It therefore provides a spatially resolved metric of the mixed *a*/*c* Raman response across the domain wall. Fig. S3a,b illustrates the construction of the DWi maps for the zero-external-power reference and for an illumination power of 100 mW. The stability of the spectral-unmixing procedure was evaluated from the residual error returned by the linear least-squares decomposition. For all analysed datasets, this residual remained below approximately 10^{-3} of the normalized Raman signal, indicating that the separation of the *a*- and *c*-domain spectral contributions is highly stable. The uncertainty associated with the spectral decomposition is therefore not a dominant contribution to the variability of the DWi-derived interfacial width and was not introduced as an additional independent term in the subsequent uncertainty propagation.

For each optical power, ten DWi profiles were extracted at different positions along the same domain-wall segment for both the off and on states, as illustrated in Fig. S3c,d. The profiles were extracted perpendicular to the local wall orientation after applying the geometrical correction described in Section S1. Each profile was independently fitted using a symmetric Gaussian function with a constant offset, and the effective Raman-resolved interfacial width was obtained from the corresponding full width at half maximum (FWHM). The fitting uncertainty associated with each individual FWHM was obtained from the covariance matrix of the nonlinear least-squares fit.

The ten FWHM values obtained for each experimental condition were subsequently used to evaluate the spatial variability of the Raman-resolved interfacial width along the investigated wall segment.

The mean FWHM and corresponding standard deviation (SD) were calculated for each optical power and illumination state. The SD, rather than the standard error of the mean, was retained as a conservative measure of the experimental variability because the ten profiles originate from different positions within the same Raman map and therefore do not constitute independent experimental replicates.

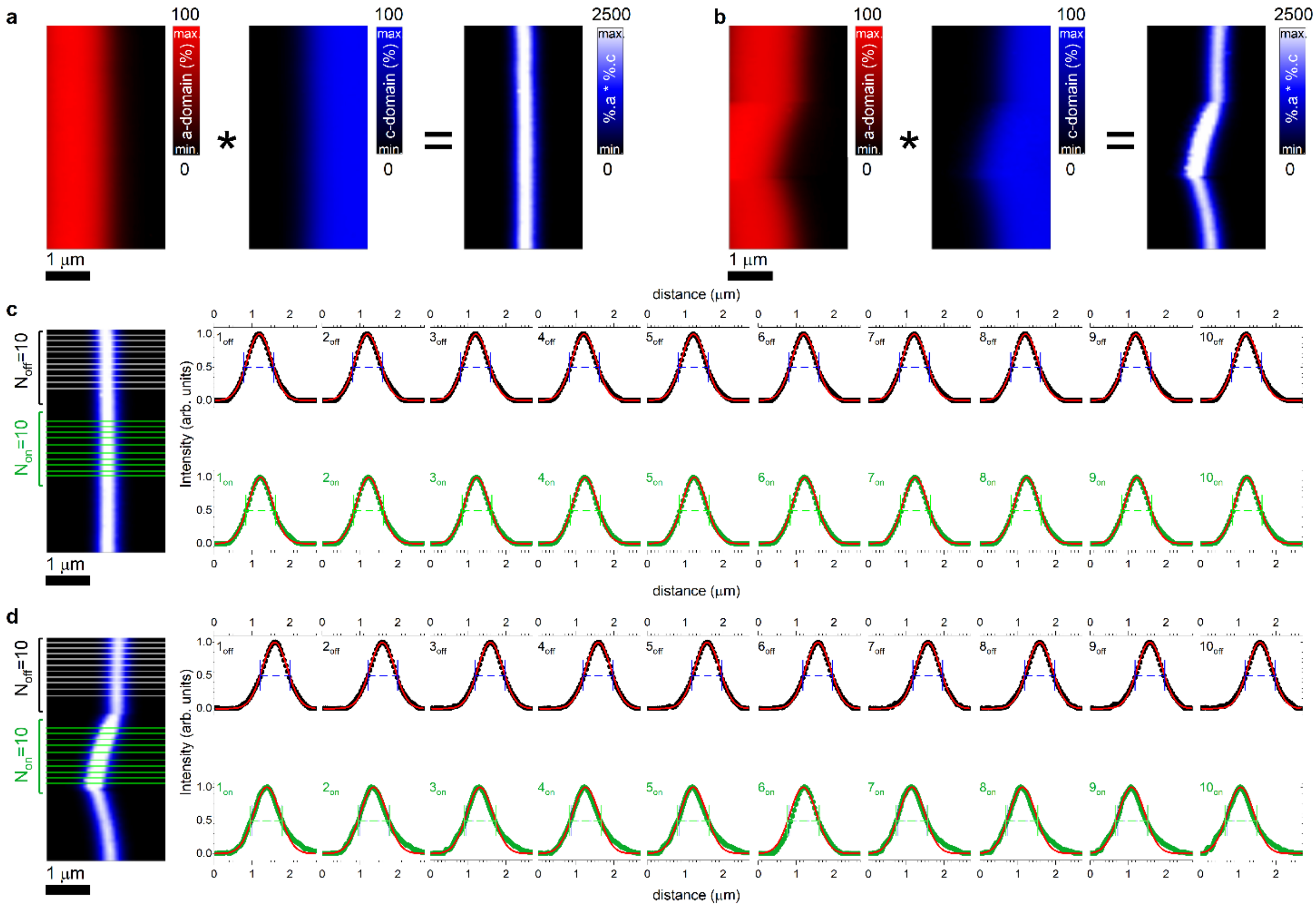

Figure S3 | Determination of the effective Raman-resolved interfacial width. a,b, Construction of the two-dimensional domain-wall index (DWi) maps from the relative *a*- and *c*-domain spectral contributions (%a and %c), illustrated for the zero-external-power reference and for an illumination power of 100 mW, respectively. The DWi is obtained from the product of both contributions and highlights the spatial region in which the Raman signatures of the adjacent *a*- and *c*-domains coexist. **c,d,** Extraction and fitting of ten DWi profiles at different positions along the same domain-wall segment for the corresponding off and on states at 0 and 100 mW. Experimental profiles were independently fitted using a symmetric Gaussian function with a constant offset, and the effective Raman-resolved interfacial width was determined from the fitted FWHM. The same procedure was applied to all illumination powers and the complete results are summarized in Table S1.

For each optical power, the corresponding off state was used as the experimental reference. The absolute illumination-induced change, ΔFWHM, was obtained from the difference between the mean on and off widths, and its uncertainty was calculated by propagation of the corresponding standard deviations. The relative broadening was calculated from the on/off width variation, and its uncertainty was determined by standard propagation of the off and on width uncertainties. The individual FWHM values and their fitting uncertainties, together with the mean off and on widths, their standard deviations, and the resulting absolute and relative width changes, are summarized in Table S1. The zero-external-power control yields a relative width variation of only-0.1 ± 0.3%, consistent with no measurable change.

These results show that the illumination-induced changes exceed the experimental variability associated with the width-extraction procedure and follow a systematic power-dependent trend. The extracted FWHM should nevertheless be interpreted as an effective Raman-resolved interfacial width, rather than as the intrinsic nanometric polarization-gradient width of the ferroelectric domain wall, since the absolute Raman-resolved profile also contains contributions from the finite confocal sampling volume and the experimental geometry discussed in Sections S1 and S2.

Table S1. FWHM values and statistical analysis of the Raman-resolved interfacial width

Optical power (mW)	N_{off}	$\mathrm{FWHM}_{\mathrm{off}}$ (nm)	$\overline{\mathrm{FWHM}}_{\mathrm{off}}$ (nm)	N_{on}	$\mathrm{FWHM}_{\mathrm{on}}$ (nm)	$\overline{\mathrm{FWHM}}_{\mathrm{on}}$ (nm)	ΔFWHM (nm)	Δ domain wall width (%)
**0**	1	760.5 ± 6.4	**763.4 ± 1.8**	1	761.9 ± 7.8	**762.7 ± 1.6**	**-0.7 ± 2.4**	**– 0.1 ± 0.3**
	2	766.2 ± 7.6		2	761.9 ± 7.6			
	3	765.6 ±7.5		3	761.3 ± 7.3			
	4	763.5 ± 7.8		4	762.7 ± 7.3			
	5	762.5± 6.5		5	761.8 ± 7.7			
	6	764.8 ± 8.4		6	764.9 ± 8.1			
	7	763.3± 7.5		7	766.2 ± 8.1			
	8	762.3 ± 7.9		8	762.2 ± 7.3			
	9	764.0 ± 9.0		9	762.3 ± 7.4			
	10	761.2 ± 8.7		10	761.6 ± 7.9			
**25**	1	769.7 ± 2.4	**775.8 ± 3.5**	1	802.6 ± 5.9	**805.7 ± 7.4**	**30.0 ± 8.2**	**3.9 ± 1.1**
	2	775.8 ± 2.6		2	811.7 ± 5.7			
	3	773.8 ± 2.5		3	811.8 ± 5.8			
	4	772.9 ± 2.8		4	805.5 ± 5.7			
	5	777.4 ± 2.5		5	791.5 ± 5.6			
	6	778.5 ± 2.5		6	815.1 ± 5.5			
	7	780.7 ± 2.6		7	798.1 ± 5.9			
	8	778.8 ± 2.9		8	806.1 ± 5.7			
	9	772.5 ± 2.7		9	813.0 ± 5.9			
	10	778.3 ± 2.6		10	802.0 ± 6.0			
**50**	1	771.7 ± 8.6	**772.8 ± 2.5**	1	800.2 ± 18.8	**812.4 ± 6.2**	**39.5 ± 6.7**	**5.1 ± 0.9**
	2	778.0 ± 9.1		2	820.5 ± 19.6			
	3	769.3 ± 8.1		3	816.4 ± 18.0			
	4	771.5 ± 8.2		4	807.5 ± 19.8			
	5	772.2 ± 8.4		5	807.5 ± 18.7			
	6	770.3 ± 7.8		6	809.7 ± 17.7			
	7	774.2 ± 9.1		7	817.0 ± 17.2			
	8	774.5 ± 9.1		8	812.2 ± 17.6			
	9	772.2 ± 7.5		9	818.2 ± 18.3			
	10	774.4 ± 6.9		10	814.2 ± 19.5			
**75**	1	764.7 ± 6.8	**759.7 ± 5.7**	1	804.2 ± 18.4	**815.7 ± 5.9**	**56.0 ± 8.2**	**7.4 ± 1.1**
	2	768.4 ± 7.0		2	823.1 ± 19.2			
	3	756.7 ± 6.5		3	819.3 ± 17.7			
	4	748.4 ± 6.0		4	810.9 ± 19.4			
	5	762.0 ± 6.6		5	811.0 ± 18.3			
	6	753.9 ± 6.7		6	813.1 ± 16.7			
	7	758.7 ± 7.4		7	819.8 ± 16.8			
	8	761.5 ± 7.0		8	815.4 ± 17.3			
	9	763.3 ± 6.8		9	821.0 ±17.9			
	10	759.2 ± 7.2		10	819.6 ± 19.1			
**100**	1	740.6 ± 18.9	**746. 5 ± 4.0**	1	817.6 ± 9.8	**819.1 ± 6.2**	**72.6 ± 7.4**	**9.7 ± 1.0**
	2	751.4 ± 20.4		2	828.9 ± 11.2			
	3	749.3 ± 24.1		3	816.3 ±12.8			
	4	751.7 ± 26.7		4	817.2 ± 13.5			
	5	746.1 ± 21.8		5	823.8 ± 11.65			
	6	742.9 ± 23.5		6	819.1 ± 10.4			
	7	748.8 ± 19.7		7	805.5 ± 12.9			
	8	742.6 ±27.1		8	817.7 ± 13.1			
	9	748.4 ± 24.8		9	821.2 ± 12.2			
	10	743.1 ± 19.1		10	823.9 ± 10.7			

S4. Raman-active modes and domain selectivity in tetragonal $BaTiO_3$

Table S2 summarizes the Raman-active modes of tetragonal $BaTiO_3$, including their symmetry assignments, characteristic Raman shifts, and domain selectivity. The experimental Raman spectra shown in Figures 2d and 2e of the main manuscript reveal distinct spectral signatures for the *a*- and *c*-domain configurations. In particular, while modes 1-6 are observed in *c*-domains, modes 4 and 6 are absent in *a*-domains. These domain-dependent spectral differences provide a robust spectroscopic fingerprint for distinguishing the two ferroelastic domain states and constitute the basis of the spectral-unmixing procedure used to determine the relative *a*- and *c*-domain contributions (%a and %c) and the Raman-derived domain-wall metric (DWi).

Table S2. Lists the Raman modes of tetragonal $BaTiO_3$, together with their symmetry assignments and the nature of each mode. In the P4mm ($C4v^1$) structure, nuclear site group analysis predicts Raman-active phonons of symmetry $3A_1 + B_1 + 4E$. Long-range electrostatic interactions split the transverse and longitudinal components of these modes, yielding $3[A_1(TO) + A_1(LO)] + B_1 + 4[E(TO) + E(LO)]$.

**Raman Shift** (cm^{-1})	**Symmetry**	**Abbreviated** number	**Active in** c-domai	**Active in** a-domai	Reference
36	E (TO)	**1**	✓	✓	1-5
170	A_1 (TO)				2-7
180	E (TO_2), E (LO)				1-5
185	A_1 (LO)				2-7
210-270	A_1 (TO_2)	**2**	✓	✓	2-7
305	E (TO_3 + LO_2)	**3**	✓	✓	1-5
305	B_1				1-5
463	E (LO_3)	**4**	✓	✗	1-5
475	A_1 (LO_2)				2-7
486	E (TO_4)				1-5
518	E (TO_5)	**5**	✓	✓	1-5
520	A_1 (TO_3)				2-7
715	E (LO_4)	**6**	✓	✗	1-5
720	A_1 (LO_3)				2-7

References

1. DiDomenico, M., Wemple, Jr., S. H., Porto, S. P. S., & Bauman, R. P. Raman Spectrum of Single-Domain $BaTiO_3$. *Phys. Rev.* **174**, 522-530 (1968).
2. Venkateswaran, Uma D., Naik, Vaman M., & Naik, Ratna. High-pressure Raman studies of polycrystalline $BaTiO_3$. *Phys. Rev.B* **58**, 14256 (1998).
3. Dobal, P. S., & Katiyar. R. S. Studies on ferroelectric perovskites and Bi-layered compounds using micro-Raman spectroscopy. *J. Raman Spectrosc.* **33**, 405-423 (2002)
4. Shiratori, Y., Pithan, C., Dornseiffer. J., & Waser. R. Raman scattering studies on nanocrystalline $BaTiO_3$ Part I – isolated particles and aggregates. *J. Raman Spectrosc.* **38,** 1288-1299 (2007)
5. Shiratori, Y., Pithan, C., Dornseiffer. J., & Waser. R. Raman scattering studies on nanocrystalline $BaTiO_3$ Part II – consolidated polycrystalline ceramics *J. Raman Spectrosc.* **38,** 1300-1306 (2007)
6. Pinczuk, A., Taylor, W. T., Burstein, E., & I. Lefkowitz, I. The Raman Spectrum of $BaTiO_3$. *Solid State Commun.* **5**, 429-433 (1967).
7. Burns, Gerald., & Scott, Bruce. A. Raman Scattering in the Ferroelectric System $Pb_{1-x}Ba_xTiO_3$. *Solid State Commun.* **9**, 813-817 (1971).
